# Engineered model of t(7;12)(q36;p13) AML recapitulates patient-specific features and gene expression profiles

**DOI:** 10.1038/s41389-022-00426-2

**Published:** 2022-09-03

**Authors:** Denise Ragusa, Ylenia Cicirò, Concetta Federico, Salvatore Saccone, Francesca Bruno, Reza Saeedi, Cristina Sisu, Cristina Pina, Arturo Sala, Sabrina Tosi

**Affiliations:** 1 College of Health, Medicine and Life Sciences, Division of Biosciences, Brunel University London, Uxbridge, UB8 3PH UK; 2 Centre for Genome Engineering and Maintenance (CenGEM), Brunel University London, Uxbridge, UB8 3PH UK; 3 Leukaemia and Chromosome Research Laboratory, College of Health, Medicine and Life Sciences, Brunel University London, Kingston Lane, Uxbridge, UB8 3PH UK; 4 Centre for Inflammation Research and Translational Medicine (CIRTM), Brunel University London, Uxbridge, UB8 3PH UK; 5 Department of Biological, Geological and Environmental Sciences, University of Catania, Via Androne 81, 95124 Catania, CT Italy

**Keywords:** Cancer genetics, Leukaemia

## Abstract

Acute myeloid leukaemia carrying the translocation t(7;12)(q36;p13) is an adverse-risk leukaemia uniquely observed in infants. Despite constituting up to 30% of cases in under 2-year-olds, it remains poorly understood. Known molecular features are ectopic overexpression of the *MNX1* gene and generation of a fusion transcript in 50% of patients. Lack of research models has hindered understanding of t(7;12) biology, which has historically focused on *MNX1* overexpression rather than the cytogenetic entity itself. Here, we employed CRISPR/Cas9 to generate t(7;12) in the human K562 cell line, and in healthy CD34+ haematopoietic progenitors where the translocation was not sustained in long-term cultures or through serial replating. In contrast, in K562 cells, t(7;12) was propagated in self-renewing clonogenic assays, with sustained myeloid bias in colony formation and baseline depletion of erythroid signatures. Nuclear localisation analysis revealed repositioning of the translocated *MNX1* locus to the interior of t(7;12)-harbouring K562 nuclei — a known phenomenon in t(7;12) patients which associates with ectopic overexpression of *MNX1*. Crucially, the K562-t(7;12) model successfully recapitulated the transcriptional landscape of t(7;12) patient leukaemia. In summary, we engineered a clinically-relevant model of t(7;12) acute myeloid leukaemia with the potential to unravel targetable molecular mechanisms of disease.

## Introduction

The t(7;12)(q36;p13) translocation is a recurrent chromosomal rearrangement uniquely associated with infant acute myeloid leukaemia (AML) [1, 2]. T(7;12) is the second most common cytogenetic abnormality in AML patients below the age of 2 years [3], and associates with a dismal prognosis. Structurally, t(7;12) is a balanced translocation disrupting the long arm of chromosome 7 (7q36.3) and the short arm of chromosome 12 (12p13.2), producing two derivative chromosomes, der(7) and der(12). The 7q36.3 breakpoint lies proximal to the *MNX1* gene, which is entirely relocated to der(12). The breakpoint on 12p13, on the contrary, disrupts the *ETV6* gene on its 5’ portion [4–6].

Mechanisms of leukaemogenesis associated with t(7;12) remain poorly understood. A *MNX1/ETV6* fusion mRNA transcript is produced from der(12) in approximately half of patients with t(7;12) [7–9], but a translated MNX1/ETV6 protein has never been detected. Introduction of the chimaeric transcript did not transform mouse bone marrow cells, suggesting that it may not contribute to the leukaemic phenotype [10].

A common feature among all t(7;12) patients is overexpression of the *MNX1* gene [7, 9, 11], which is proposed to result from disruption of its genomic positioning [7]. The majority of reports on t(7;12)-mediated leukaemogenesis have focused on *MNX1* overexpression [10, 12, 13]. In vitro, overexpression of *MNX1* in mouse haematopoietic stem cells (HSCs) did not result in expansion or increased survival capacity [10], and was shown to induce senescence [13]. In human cord blood CD34+ haematopoietic stem and progenitor cells (HSPC), *MNX1* overexpression induced erythroid transcriptional programmes and reduced colony-forming capacity. In vivo, transplantations of *MNX1* overexpressing bone marrow cells did not cause leukaemia in the recipients, but resulted in an accumulation of *MNX1*-overexpressing cells in the megakaryocytic-erythrocyte fraction, but not in the granulocytic-monocyte nor in the mature lymphoid compartments [13].

Genome editing technologies can be harnessed for generation of chromosomal abnormalities [14–16]. The CRISPR/Cas9 gene editing tool employs the endonuclease activity of the Cas9 enzyme to induce DNA double stranded breaks (DSB) at specific loci directed by guide RNAs (gRNA). The induction of two simultaneous DSBs can lead to erroneous break repair and rejoining of the ‘wrong’ DNA ends, thereby forming a chromosomal translocation [17, 18]. Here, we sought to recreate the t(7;12) rearrangement in vitro using CRISPR/Cas9 in the attempt to uncover biological cues to its mechanisms in AML.

## Results and discussion

**Fig. 1:**
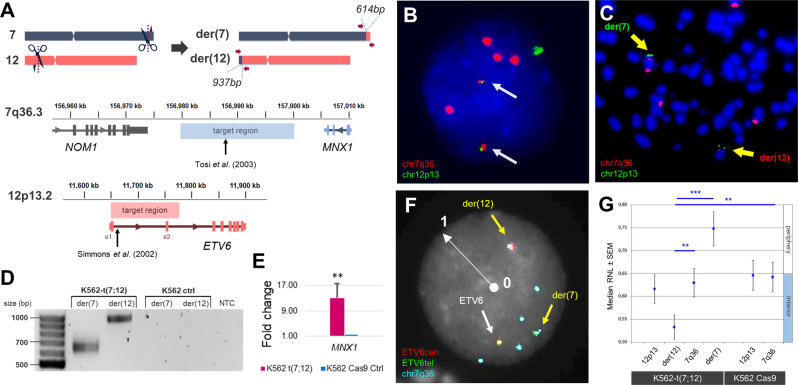
Generation of t(7;12)(q36;p13) in K562 cells. **A** Schematic representation of target regions for CRISPR/Cas9-directed cleavage on chromosomes 7 and 12 used for guide RNA (gRNA) design. Fusion junctions are flanked by arrows representing PCR primers used for confirmation, yielding products of 614 and 937 bp. Below, detailed location of the targeted regions on chromosome 7q36.3 and 12p13.2 with reference to known t(7;12) breakpoints used to generate the translocation. **B** FISH using the specific t(7;12) probe XL t(7;12) MNX1/ETV6 (MetaSystems Gmbh, Altlussheim, Germany, Supplementary Table 3) hybridising chromosome 7q36 in red and chromosome 12p13 in green. Two yellow fusion signals, pointed by arrows, indicate the presence of t(7;12) in a representative interphase nucleus of K562-t(7;12). The karyotype of K562 is nearly tetraploid and harbours complex rearrangements, including a duplications of the 7q36 locus within the short arm of chromosome 7, hence showing five 7q36 signals and two 12p13 signals [31] (Supplementary Fig. 1). **C** Metaphase spread of K562-t(7;12) hybridised with the same probe shows the presence of derivative chromosomes der(7) and der(12), pointed by arrows. **D** Confirmation of the presence of t(7;12) in K562-t(7;12) but not K562 control (‘ctrl’) cells (K562 electroporated with Cas9 only) by PCR amplification of fusion junctions and product separation on agarose gel; bands correspond to the predicted sizes shown in **A**. NTC no template control. **E** qRT-PCR validation of overexpression of *MNX1* in K562-t(7;12) compared to K562 control. The fold change was calculated using the ΔΔCt method by normalisation to the endogenous gene *HPRT1*. Error bars represent standard deviation (SD) of *n* = 3. Primers are reported in Supplementary Table 4. **F** A custom-made 3-colour probe (MetaSystems dual-colour ETV6 + PAC-derived RP5-1121A15) hybridising *ETV6* portions in red (centromeric) and green (telomeric), and the *MNX1* locus in cyan (Supplementary Fig. 1 and Supplementary Table 3), allowed visualisation of both derivative chromosomes in interphase nuclei (pointed by yellow arrows). The white radius arrow represents the distance between nuclear interior (value = 0) and nuclear periphery (value = 1), which was used in the calculation of radial nuclear locations (RNL). **G** RNL of der(7) and der(12) signals in K562-t(7;12) interphase nuclei. The RNL values are expressed as median values of individual localisations of FISH signals (200 nuclei analysed per condition). Errors bars represent standard error of the mean (SEM). Values closer to 0 indicate an internal position within the nucleus (described in detail in Federico et al. [32]).

By delivery of CRISPR/Cas9 ribonucleoprotein (RNP) complexes targeting clinically accurate breakpoints described by Tosi et al. [5] and Simmons et al. [4] (Fig. 1A and Supplementary Table 1), we were able to achieve the t(7;12) rearrangement in the leukaemia cell line K562, as confirmed by fluorescence in situ hybridisation (FISH) (Fig. 1B, C and Supplementary Fig. 1) and amplification of genomic fusion junctions by polymerase chain reaction (PCR) (Fig. 1D and Supplementary Fig. 2). CRISPR/Cas9 editing via RNP complex electroporation allowed prompt activity without persistent vector presence, as well as a more physiological recapitulation of DSB generation leading to the formation of chromosomal translocations [14–18]. Consistent with only a fraction of cells undergoing illegitimate repair of the breaks to form the rearrangement, the frequency of t(7;12) formation was estimated at 1% by FISH (Supplementary Fig. 1G). Homogeneous t(7;12)-harbouring lines (K562-t(7;12)), as well as controls (K562-Ctrl), were obtained by single cell cloning by limiting dilution and screened by PCR, from which three clones were derived by subcloning (Supplementary Fig. 3).

While *MNX1* is already expressed in K562 cells [19], the translocation further upregulated its expression (Fig. 1E), consistent with ectopic *MNX1* overexpression in patients [7, 9, 11]. Altered nuclear positioning has been proposed as a significant mechanism of *MNX1* overexpression in t(7;12) leukaemia, with relocalisation of the der(12) containing the translocated *MNX1* gene to a more internal transcriptionally active position within the nucleus, where expression of *MNX1* can be triggered [7]. We used nuclear localisation analysis to understand whether this phenomenon was recapitulated in the K562 model. FISH using a custom 3-colour probe allowed to distinguish both derivatives in interphase nuclei (Fig. 1F and Supplementary Fig. 1D–F) in order to calculate radial nuclear location (RNL) values for each locus with respect to their positioning towards the interior or peripheral nuclear space (Fig. 1F, G). As described in patients, the introduction of t(7;12) in K562 cells resulted in the altered positioning of the der(12) towards the nuclear interior, consistent with the relative gain in *MNX1* expression (Fig. 1E). Conversely, the der(7) containing the 5’ of *ETV6* was repositioned towards the periphery (Fig. 1G), also in agreement with the patient analysis by Ballabio et al. [7]. Nuclear organisation as a mechanism of regulation of *MNX1* expression was also identified in leukaemias harbouring different interstitial deletions of chromosome 7q [20]. Specifically, the localisation of *MNX1* was shown to be dependent on the GC-content of the chromosomal band harbouring the proximal breakpoint, with *MNX1* being expressed when breakpoints affected GC-rich bands, which resulted in relocation of *MNX1* towards the nuclear interior; in contrast, GC-poor breakpoints resulted in localisation at the periphery and *MNX1* was not expressed [20]. The gene repositioning caused by the genetically engineered translocation mimics the observations in patient cell nuclei, and captures a representative feature of t(7;12) [7]. The translocation changes the regulatory context of *MNX1* and results in its ectopic expression and nuclear repositioning, but the relative causality of the two observations cannot be definitively established. Future local and global analysis of *MNX1* genomic interactions through high resolution chromatin capture methods may shed light on the order of events, as well as help establish the functional role of existing and de novo interactions on leukaemogenesis driven by the t(7;12) rearrangement [21, 22].

**Fig. 2:**
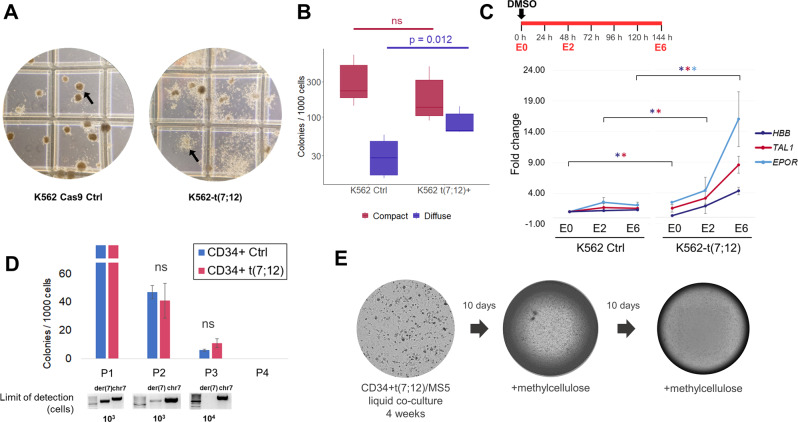
Functional effects of t(7;12) in haematopoietic cells. **A** Representative images of colony-forming assays of K562 control (‘K562 Cas9 Ctrl’) (left) and K562-t(7;12) (right) on Methocult H4434 Classic (StemCell Technologies). Arrows indicate distinct morphologies of compact (left) and diffuse colonies (right). **B** Frequency of colony formation per 1000 K562 Cas9 Ctrl and K562-t(7;12) plated cells. Mean and 95% CI of *n* = 3 independent experiments. Statistical significance determined by two-tailed Student’s *t* test. **C** Quantitative RT-PCR gene expression analysis of erythroid differentiation of K562-t(7;12) cells; schematic representation of differentiation assay (top left). In total, 100,000 cells were plated in RPMI medium supplemented with foetal bovine serum (FBS) with 1.5 μM DMSO added at time point 0 h. ‘E’ indicates the day of erythroid differentiation, from E0 (0 h) to E6 (144 h). Expression of erythroid markers *EPOR*, *TAL1*, and *HBB* was quantified in K562-t(7;12) through E0 to E6, calibrated to K562-Ctrl E0. Fold changes were calculated using the ΔΔCt method by normalisation to the endogenous gene *HPRT1*. Asterisks indicate statistically significant fold changes of K562-t(7;12) compared to K562 control for each gene. Full statistical tests on all conditions are shown in Supplementary Fig. 4. Primers are reported in Supplementary Table 4. **D** Introduction of the t(7;12) translocation in healthy CD34+ HSPCs enriched from mobilised peripheral blood by magnetic separation. Serial replating colony-forming assays in Methocult H4434 Classic medium with multi-lineage cytokines. In total, 10,000 CD34+ HSPCs were plated in methylcellulose-based medium and allowed to form colonies. Colonies were scored, collected and dissociated after each plating, and replated over several rounds. HSPCs are expected to originate progressively less colonies, unless a survival advantage is gained through transformation. ‘P1–P4’ indicate the order of plating. Progressively less colonies were produced, without statistically significant differences between the two conditions. Agarose gel electrophoresis of semi-quantitative PCR is shown underneath each colony count, indicating the limit of detection of the der(7) fusion junction. A non-translocated region of chromosome 7 was used as a control (‘chr 7’) to confirm the presence of amplifiable template DNA. **E** MS5 stroma with the addition of CD34+ t(7;12) cells. Two colonies were visible following methylcellulose addition after 4 weeks of co-culture. No colonies visible following a second round of co-culture and methylcellulose addition.

While the transformed nature of the K562 cell line did not allow assessment of the transforming capacity of t(7;12) in this system, we observed a distinct phenotype associated with the K562-t(7;12). In clonogenic colony-forming assays on methylcellulose-based medium, K562-t(7;12) produced a significantly higher proportion of colonies with a diffuse phenotype, at the expense of the more prevalent compact colonies of K562 control (Fig. 2A, B). K562 produces mostly erythroid-like compact colonies, while the diffuse phenotype associates with an immature myeloid identity (CFU-G or CFU-GM-like) [23], suggesting that t(7;12) changes the erythroid differentiation bias of K562. Importantly, the phenotype was maintained through replating, indicating that the translocation can be perpetuated through self-renewal. This is in contrast with the effects of *MNX1/ETV6* and *MNX1* overexpression in adult haematopoietic tissues, for which phenotypic changes could not be maintained through self-renewal [10, 13].

We further confirmed the depletion of erythroid identity by subjecting both K562-t(7;12) and K562 control to induced erythroid differentiation assay by adding DMSO to the culture medium and assessing the expression of erythroid marker genes [24] (Fig. 2C and Supplementary Fig. 4). At the beginning of the assay at erythroid day 0 (E0), we observed a lower expression of *HBB* and *TAL1* in K562-t(7;12) compared to K562 control (Supplementary Fig. 4A), indicating an attenuation of erythroid signatures, compatible with the myeloid bias of K562-t(7;12) colony formation. Control K562 underwent differentiation and plateaued at E2, as seen by upregulation of *HBB, TAL1*, and *EPOR*. In K562-t(7;12), the differentiation capacity was not blocked by the presence of the translocation as seen by the upregulation of the three genes, albeit displaying higher fold changes compared to the control (Fig. 2C and Supplementary Fig. 4). In this light, the erythroid differentiation assay confirmed that the erythroid differentiation programme was still achievable in K562-t(7;12), but at higher fold gene expression changes, suggesting retainment of erythroid cells at an earlier differentiation state in the absence of external differentiation cues, and/or relative expansion of myeloid-affiliated cells as a result of the translocation. Together with the colony-forming capacity suggesting an imbalance between erythroid and granulocytic/monocyte-like colonies (Fig. 2A), we demonstrate that t(7;12) can affect erythroid differentiation programmes. In earlier models of t(7;12), *MNX1*-overexpressing bone marrow cells transplanted into recipient mice were primarily found in myeloid-committed progenitor populations and particularly within megakaryocytic/erythroid progenitors [13]. In human cord-blood CD34+ HSCPs, *MNX1* overexpression induced erythroid transcriptional programmes and reduced overall colony-forming capacity [13], indicating that t(7;12) encompasses but may exceed the observed effects of *MNX1* overexpression on erythroid differentiation.

In order to understand t(7;12) effects in untransformed haematopoietic cells, we introduced the translocation in adult CD34+ HSCPs. Despite being able to confirm its presence by PCR (Supplementary Fig. 5A), the frequency of t(7;12)-harbouring cells decreased through serial replating in methylcellulose-based colony-forming assays (Fig. 2D), as well as in liquid culture (Supplementary Fig. 5B), as determined by semi-quantitative PCR. We used long-term culture initiating cell (LTC-IC) assays to test for leukaemia-initiating effect. This two-step assay involves a 4-week co-culture with MS5 stroma, which selects for self-renewing or slowly proliferating HSPCs, followed by assessment of HSPC functional potential in colony-forming assays. Similarly to short-term clonogenic assays, edited CD34+ cells did not show any advantage in persistence of LTC-IC, as measured by the lack of sustained colony formation following co-culture in MS5 and serial methylcellulose replating (Fig. 2E). Taken together, our observations are in line with the previously reported inability of *MNX1* overexpression to transform adult haematopoietic cells [10, 13]. This may suggest a requirement for additional genetic events to achieve full *MNX1*/t(7;12)-associated transformation, or otherwise suggest that t(7;12) selectively affects a cell type or developmental stage not efficiently represented in adult mouse or human haematopoietic tissues. The transformed nature of K562 cells, with or without contribution from the persistence of foetal developmental programmes, as evidenced by the nature of haemoglobins expressed [25], may provide a more suitable substrate for persistence of t(7;12).

**Fig. 3:**
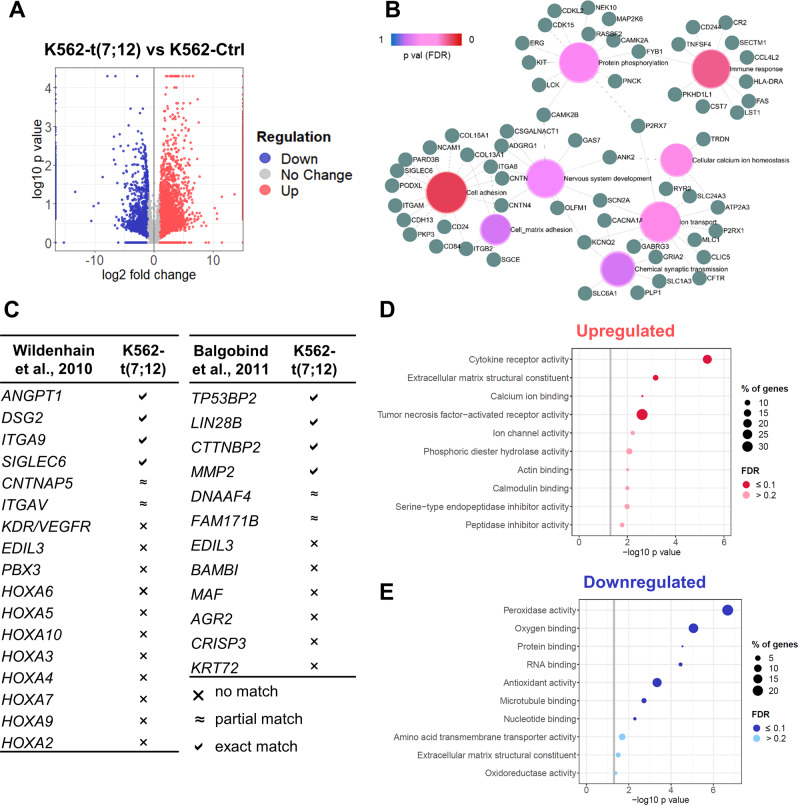
Transcriptional analysis of K562-t(7;12). **A** Volcano plot showing differentially expressed genes by RNA sequencing of K562-t(7;12) compared to K562-Ctrl (three subclones per condition), depicting up- (in red) and downregulated (in blue) genes and their statistical significance by −log10 *p* value. Genes with an absolute log2 fold change lower than 1 are shown in grey (‘No change’). **B** Gene Ontology (GO) network plot of differentially expressed genes by false discovery rate (FDR) < 0.1 showing significant biological processes (PANTHER) and genes associated to each GO term, constructed on ExpressAnalyst (available at www.expressanalyst.ca). **C** Comparison of differentially expressed genes in K562-t(7;12) and published reports of genes associated with t(7;12) patients. Symbols indicate difference in expression between K562-t(7;12) and K562 control by statistical significance (threshold *p* ≤ 0.05), defined as an exact gene match, a partial match (i.e. dysregulation of a gene belonging to the same family), or no match. GO terms relating to molecular functions (PANTHER) of upregulated (**D**) and downregulated (**E**) genes in K562-t(7;12). The grey intercept marks the *p* = 0.05 threshold in –log10. The size of the dots indicate the percentage of genes mapping to a specific term. Dark red or blue indicates an enrichment with an FDR ≤ 0.1.

In order to understand if K562-t(7;12) recapitulated molecular signatures identified in t(7;12) AML patients, we performed RNA sequencing analysis of three subclones each of K562-t(7;12) compared to K562 control, and identified 436 upregulated and 116 downregulated genes by *p* value lower than 0.01, of which 196 under the threshold of 1% false discovery rate (FDR) (Fig. 3A). Gene Ontology analysis revealed that differentially expressed genes (filtered by FDR) were involved in functions related to cell adhesion, immune response, and transport-related biological processes (Fig. 3B), as previously reported by Wildenhain et al. [10] who compared t(7;12) to *MLL* paediatric patients (Fig. 3C). Genes associated with cell adhesion and the structural component of the matrix included collagen genes (*COL*), integrins (*ITG*), and the surface markers CD24 and CD84 (Fig. 3B), which were also identified by further dissection of the upregulated genes by molecular function (Fig. 3D). Adhesion-related gene families, specifically contactins, integrins, and sialic acid-binding immunoglobulin-type lectins (Siglecs) (Supplementary Fig. 6), showed highly consistent expression profile between Wildenhain’s and our K562-t(7;12) data, with 50% of matching upregulated genes (Fig. 3C). Indeed, the involvement of cell adhesion and extracellular matrix modelling in bone marrow niche interactions has been proposed as an important leukaemic mechanism of t(7;12) [10, 12, 13, 26]. Balgobind et al. [27] also described a distinctive gene expression signature of t(7;12) leukaemia compared to a broader set of AML subtypes including *MLL* rearrangements, t(8;21), inv(16) and t(15;17), identifying 12 discriminative genes (Fig. 3C). Our model matched a differential expression of *TP53BP2, LIN28B, CTNNBP2*, and *MPP2*, capturing the Balgobind signature by matched or partially matched genes (Fig. 3C and Supplementary Fig. 6). In summary, our data provides a model for future studies of individual and coordinated gene contributions to t(7;12) biology.

Downregulated genes in K562-t(7;12) were enriched for molecular functions of macromolecule binding (Fig. 3E). Several haemoglobin genes were found to be downregulated (Supplementary Fig. 7), which are related to as functional categories of haptoglobin, haemoglobin, and oxygen binding processes (Fig. 3E). The concomitant downregulation of peroxiredoxins also highlighted peroxidase and antioxidant activities, which are consistent with biological functions of erythrocytes, and therefore accompany the relative depletion of erythroid identity (Fig. 2A–C). The signature from Wildenhain et al. [10] also included the downregulation of *HOXA* genes in t(7;12) compared to *MLL* patients, which we did not find as differentially expressed in K562-t(7;12) (Supplementary Fig. 6). This reflects the specificity of *HOXA* dysregulation in *MLL* leukaemia [28], but not necessarily the relevance in t(7;12).

**Fig. 4:**
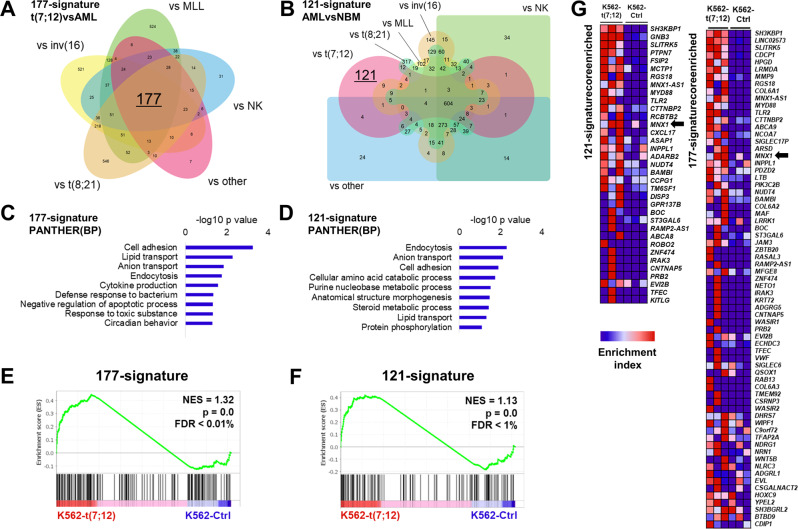
Comparison of K562-t(7;12) transcriptional landscape with t(7;12) patient signatures. **A** 177-gene signature of t(7;12)-patient gene expression extrapolated from published microarray and RNA sequencing datasets by comparison with other paediatric AML subtypes. The Venn diagram shows the 177 intersect genes. **B** 121-gene t(7;12)-specific signature inferred by comparisons of gene expressions of paediatric AML and normal bone marrow (NBM) samples. Edward’s Venn diagram highlight the 121 exclusive genes to t(7;12). **C**, **D** Gene Ontology analysis of the 177-signature and 121-signature by the PANTHER annotation repository of biological processes (BP). **E**, **F** GSEA enrichment plots of K562-t(7;12) gene expression profile using the 177- and 121-signatures. NES normalised enrichment score, FDR false discovery rate. **G** Core enriched genes from the GSEA using the 177- and 121-signatures, shown by their enrichment index in K562-t(7;12) against K562-Ctrl. *MNX1* is highlighted by the arrows.

Overall, observed gene expression changes were consistent with individual gene signatures reported in analyses of t(7;12) patients (Fig. 3C). We sought to further validate the clinical relevance of the K562-t(7;12) model by a systematic comparison with microarray and RNA sequencing data of t(7;12) patients from Balgobind et al. [27] and the TARGET database [29]. We derived a unique 177-gene signature by comparison of t(7;12) with other individual paediatric subtypes (inv(16), *MLL* rearrangements, normal karyotype = NK, t(8;21), and other) by identification of the common differential gene set (Fig. 4A). Similarly, we defined a 121-gene list uniquely differentially expressed between t(7;12), but not other AML subtypes, and normal paediatric bone marrow (Fig. 4B). Consistent with previous reports [10], both gene sets were enriched for cell adhesion, cellular transport and lipid metabolism gene ontologies (Fig. 4C, D). We also note that *HOXA* genes, previously identified by comparing t(7;12) AML with *MLL* rearrangements [10], were not present in these signatures as a result of comparisons with multiple paediatric AML subtypes. Importantly, we used the 177 and 121 t(7;12) genes as custom gene sets for Gene Set Enrichment Analysis (GSEA), and showed significant enrichment in the K562-t(7;12) model (Fig. 4E, F), thus demonstrating that engineering of the translocation in K562 cells resulted in a clinically-relevant model. Extraction of the core enriched genes, or leading edge, of GSEA identifies individual patient-specific genes recapitulated in our model (Fig. 4G), which can be regarded as future targets in mechanistic studies of t(7;12)-associated phenotypes. Crucially, *MNX1* was identified as a leading-edge gene, asserting its centrality to t(7;12) biology (Fig. 4G).

During the preparation of this manuscript, another group successfully recreated the t(7;12) translocation in human induced pluripotent stem cell (iPSC) using CRISPR/Cas9 [30], with comparable features to our model. The authors observed an increased frequency of erythroid and myeloid progenitors in colony-forming assays, coherent with an increase in myeloid transcriptional programmes. Notably, the iPSC model reproduced the overexpression of *MNX1* and production of a *MNX1/ETV6* fusion transcript, albeit at a low transcriptional level that questions its biological relevance. Despite extensive FISH analyses in early studies [4–6], the exact breakpoints required to produce the fusion transcripts from t(7;12) have not been elucidated. In Nilsson et al. [30], the expression of the *MNX1/ETV6* mRNA was achieved by targeting a similar genomic region to us on 7q36, but a distinct intronic region of *ETV6*. In our K562 model, a chimaeric transcript was not detected by potential gene fusion search by TopHat-Fusion on the RNA sequencing reads; an interesting observation given the 50% occurrence of chimaeric transcripts in patients [2]. With the continued development of genome editing technologies, we foresee that these in vitro models will allow a more precise dissection of the breakpoint regions required for the formation of the yet uncharacterised *MNX1/ETV6* chimaera. Both the iPSC and K562 models of t(7;12) showed similarities to patient-specific gene expression patterns, which strengthens the validity of CRISPR/Cas9 to generate disease models based on clinical genomic information.

In summary, we generated cellular model of t(7;12) translocation which recapitulates *MNX1* overexpression and captures cell adhesion signatures putatively core to t(7;12) oncogenic programmes. The model displays a granulocytic-monocytic phenotype compatible with the lineage affiliation of clinical t(7;12) AML, and disrupts erythroid lineage programmes as described in *MNX1* overexpression. We confirmed the inability of t(7;12) to transform adult haematopoietic tissues, highlighting the requirement for additional oncogenic hits, or a specific cellular background which may explain the unique association of t(7;12) with infants. Complementarily to earlier *MNX1*-based models [10, 13], and more recent genome edited tools [30], our t(7;12) K562 model will provide a framework for the molecular elucidation of this unique subtype of infant AML.

## Supplementary information

Supplementary material

## Data Availability

The RNA sequencing datasets generated and analysed during the current study have been deposited in the ArrayExpress repository under accession number E-MTAB-11851. The results published here are partly based upon data generated by the Therapeutically Applicable Research to Generate Effective Treatments (TARGET) (https://ocg.cancer.gov/programs/target) initiative, of the Acute Myeloid Leukemia (AML) cohort phs000465. The data used for this analysis are available at https://portal.gdc.cancer.gov/projects.
