# Supplementary material for: Engineered model of t(7;12)(q36;p13) AML recapitulates patient-specific features and gene expression profiles
Source: Oncogenesis. 2022 Sep 3;11(1):50. doi: 10.1038/s41389-022-00426-2 (PMC9440899; doi:10.1038/s41389-022-00426-2)
Supplement: Supplementary file 1 — Supplementary material [file 41389_2022_426_MOESM1_ESM.docx]

**Supplementary information**

**Engineered model of t(7;12)(q36;p13) AML recapitulates patient-specific features and gene expression profiles**

Denise Ragusa, Ylenia Cicirò, Concetta Federico, Salvatore Saccone, Francesca Bruno, Reza Saeedi, Cristina Sisu, Cristina Pina, Arturo Sala, Sabrina Tosi

**Supplementary Figures**

Supplementary Figure 1 – FISH on K562-t(7;12) and K562 control nuclei using two probe sets.

Supplementary Figure 2 – Sanger sequencing of der(7) and der(12) fusion junctions.

Supplementary Figure 3 – Agarose gel electrophoresis of direct PCR screening of single cell cloned edited K562 cells.

Supplementary Figure 4 – Erythroid differentiation assay of K562-t(7;12).

Supplementary Figure 5 – PCR amplification of fusion junctions of t(7;12) in edited CD34+ HSCPs.

Supplementary Figure 6 – Expression differences in K562-t(7;12) compared to K562 control of known t(7;12)-associated genes.

Supplementary Figure 7 – Differentially expressed haemoglobin genes in K562-t(7;12).

**Supplementary Tables**

Supplementary Table 1 – gRNA sequences

Supplementary Table 2 – PCR primer sequences

Supplementary Table 3 – FISH probes ideograms

Supplementary Table 4 – qPCR primer sequences

Supplementary Table 5 – 122-signature gene list

Supplementary Table 6 – 177-signature gene list

**Methods**

1. Cell cultures
2. CRISPR/Cas9 editing and clone selection
3. Amplification of t(7;12) fusion junctions by polymerase chain reaction (PCR)
4. Fluorescence in situ hybridisation (FISH)
5. RNL analysis
6. Real-time quantitative PCR (qPCR)
7. Colony forming assay
8. Long-Term Culture Initiating Cell Assay (LTC-IC)
9. Erythroid differentiation assay
10. RNA sequencing analysis
11. Gene Ontology analysis
12. Flow cytometry staining
13. Statistical analysis and visualisation
14. Patient signature methods and data availability
15. Gene Set Enrichment Analysis (GSEA)

**Supplementary References**

**Supplementary Figures**

**
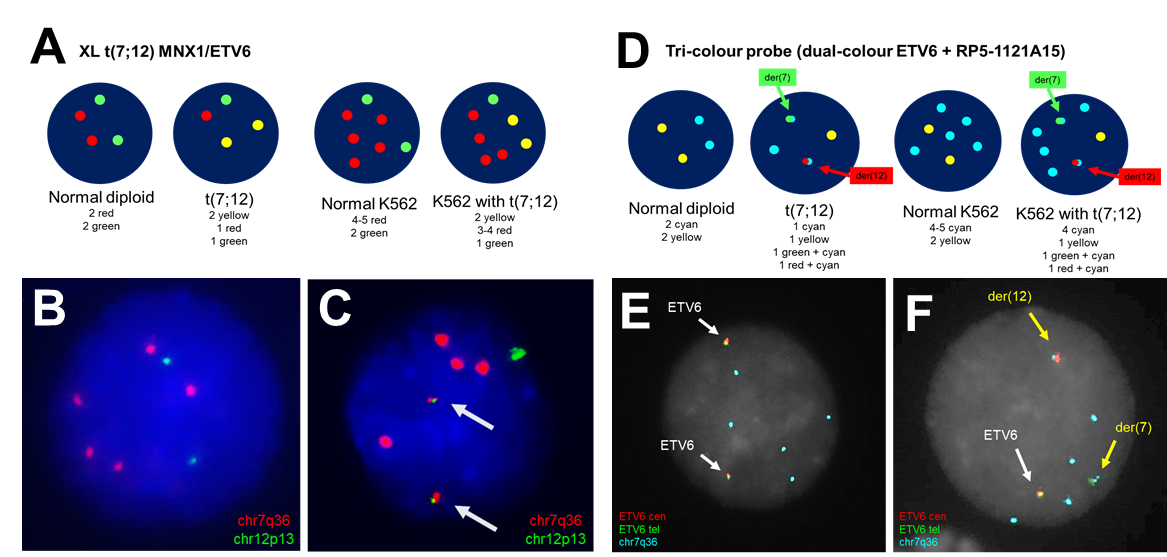
**


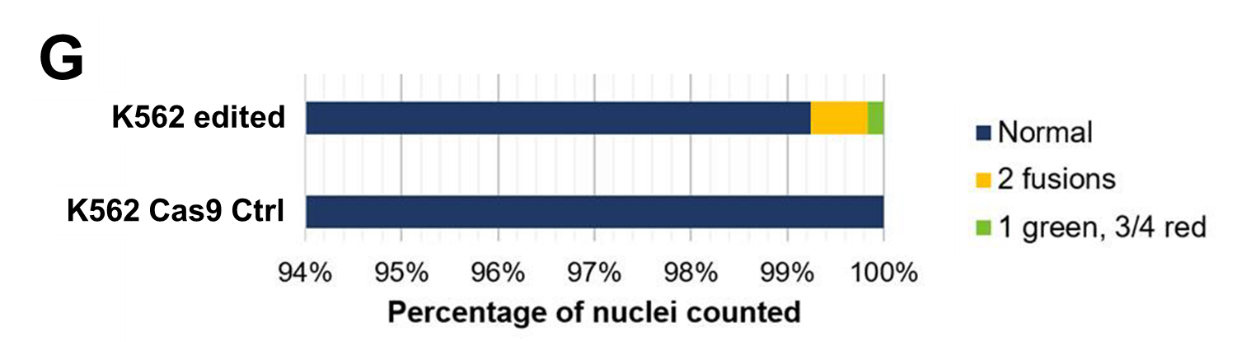


**Supplementary Figure 1 – FISH on K562-t(7;12) and K562 control nuclei using two probe sets. A)** The t(7;12)-specific FISH probe XL t(7;12) MNX1/ETV6 (MetaSystems) patterns in normal diploid nuclei and t(7;12) on the left, and normal K562 and K562 with t(7;12) on the right. The presence of the t(7;12) is confirmed by two yellow fusion signals, by splitting of the red and green signals due to the translocation. The karyotype of K562 is nearly tetraploid and harbours complex rearrangements, including a duplications of the 7q36 locus containing MNX1 within the short arm of chromosome 7, hence showing five 7q36 signals and two 12p13 signals (1) **B)** A normal pattern indicates the expected signal pattern in non-edited K562, with 5 red signals corresponding to the 7q36 locus, and 2 green signals hybridising to 12p13. **C)** Two yellow fusion signals, pointed by arrows, are indicative of the presence of the t(7;12). **D)** Tri-colour probe (dual colour ETV6 probe + RP5-1121A15) patterns in normal diploid nuclei and t(7;12) on the left, and normal K562 and K562 with t(7;12) on the right. The two derivatives are distinguishable by the fusion of the green and cyan (der7), and red and cyan (der12). **E)** Representative nucleus and metaphase of K562 Cas9 Ctrl showing the hybridisation patterns of the 3-colour probe with non-translocated ETV6 and 7q36 regions, indicated by 2 yellow fusion signals and 5 cyan signals, respectively. **F)** Representative K562-t(7;12) nucleus showing the presence of two derivative chromosomes indicated by yellow arrows, the non-translocated ETV6 allele (white arrow), and 4 non-translocated 7q36 signals. The der(7) is characterised by the fusion of the green and cyan signals, while the der(12) is distinguishable by red and cyan overlapping signals. **G)** Percentage of observed FISH signal patterns in edited K562 and K562 Cas9 Ctrl. At least 500 nuclei were counted per sample. The “2 fusions” yellow bar indicates the signal pattern consistent with the presence of t(7;12).

**
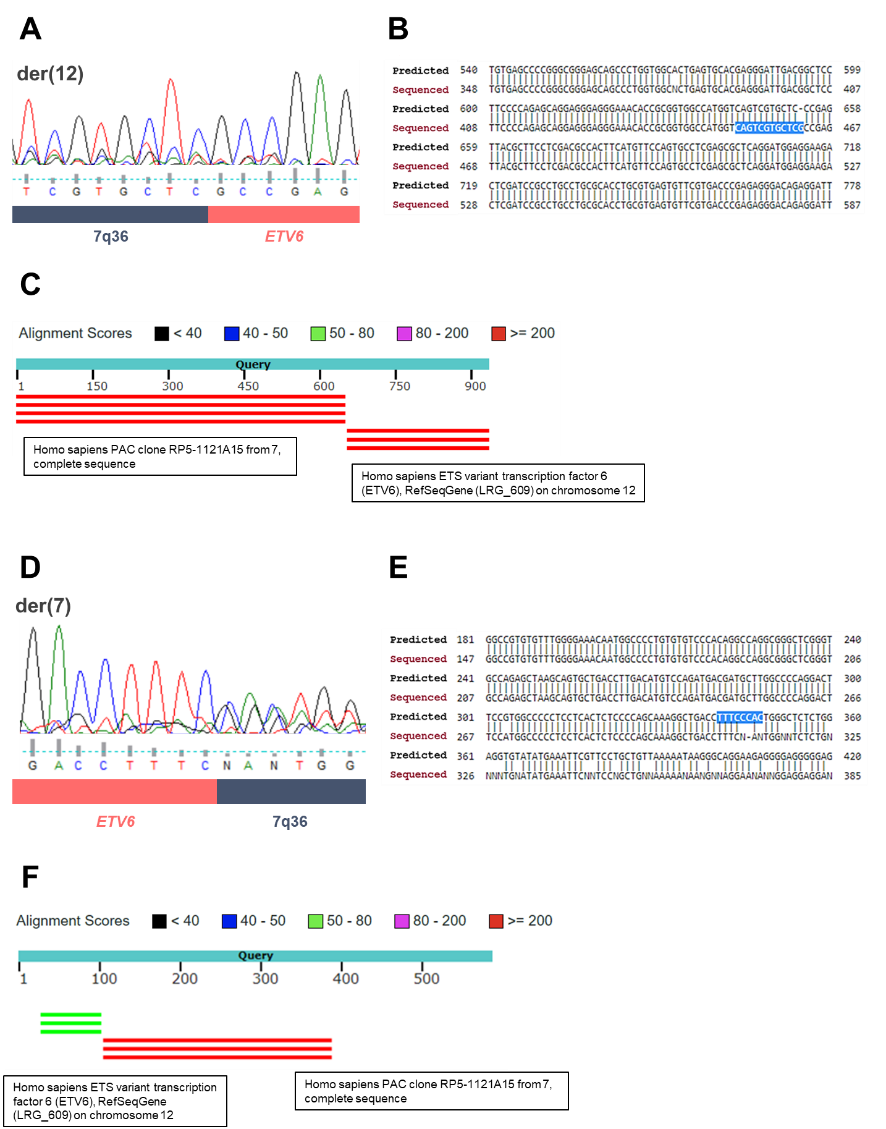
**

**Supplementary Figure 2 – Sanger sequencing of der(7) and der(12) fusion junctions.** Chromatograms showing the nucleotide sequence of amplified fusion junctions in der(12) **(A)** and der(7) **(D)**. Sequence alignments of der(12) **(B)** and der(7) **(E)** corresponded to the predicted sequence. Highlighted nucleotides in blue correspond to the fusion junction. The identity of the sequences was confirmed by BLAST, showing matching sequence identity from chromosome 7q36 region (RP5-112A15) and the ETV6 gene in both derivatives **(C,F)**.

***
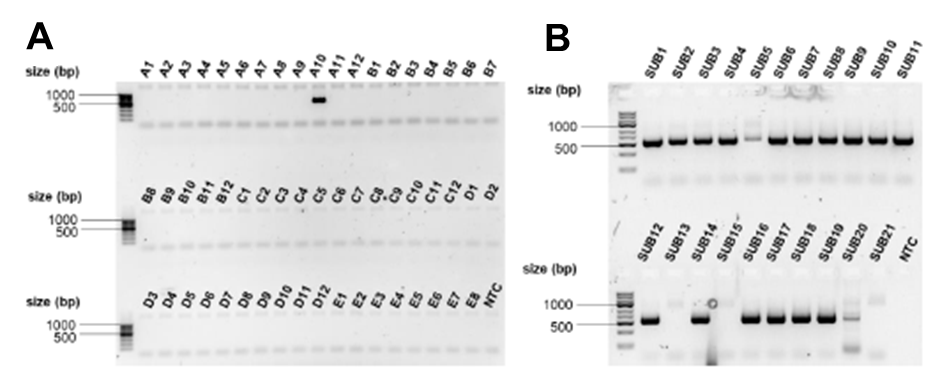
***

**Supplementary Figure 3 – Agarose gel electrophoresis of direct PCR screening of single cell cloned edited K562 cells. A)** Initial screening revealed the presence of der(7) in the clone A10, by detection of a band of 614 bp. **B)** Clone A10 was further subcloned and each subclone was screened for the presence of der(7). NTC = no template control. Molecular marker: Gene Ruler 100 bp.


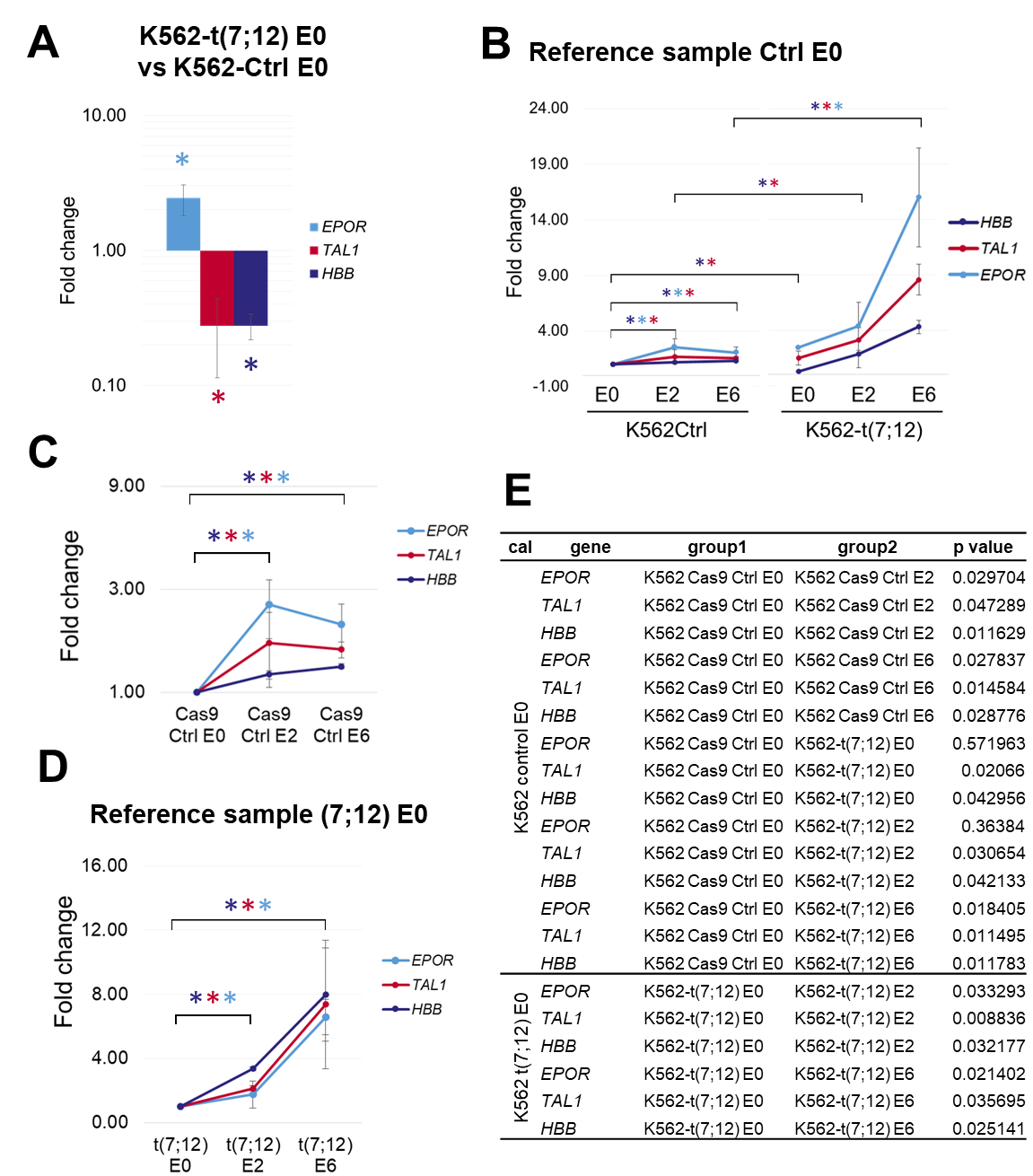


**Supplementary Figure 4 – Erythroid differentiation assay of K562-t(7;12). A)** Gene expression quantification of HBB, TAL1 and EPOR in K562-t(7;12) expressed in fold change compared to K562-Ctrl at the beginning of the erythroid differentiation assay (0 h; E0). Asterisks indicate a p value ≤ 0.05 with the colour corresponding to the gene as shown in the legend. Error bars indicate standard deviation on n=3. **B)** Full comparison of statistically significant gene expression changes, by fold changes calibrated to K562 control E0. **C)** Zoomed in view of K562 control with expanded fold change y axis. **D)** Fold changes in K562-t(7;12) calibrated to K562-t(7;12) E0, instead of K562 control. **D)** Table of p values determined by Student’s T-test in all combinations for expression changes across E0 – E2 – E6. “Cal” refers to the calibrator/reference sample of choice, either K562-Ctrl E0 or K562-t(7;12) E0. Group 1 and group 2 indicate the two conditions compared.


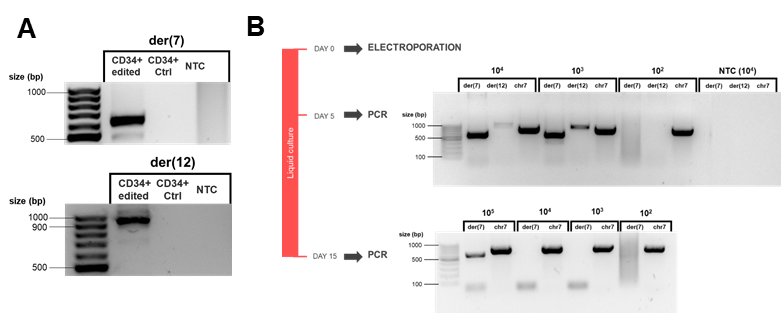


**Supplementary Figure 5 ‒ PCR amplification of fusion junctions of t(7;12) in edited CD34+ HSPCs.** Agarose gel electrophoresis of amplified fusion junctions of der(7) (expected size 614 bp) and der(12) (expected size 937 bp) Ctrl = Cas9-only Control; NTC = no template control. Molecular marker: Gene Ruler 100 bp. Following electroporation, edited CD34+ HSCPs were grown in liquid culture for 15 days and screened for the presence of t(7;12) fusion junctions by semi-quantitative direct PCR. Varying amounts of cells were directly lysed and used as template for PCR to detect der(7) (614 bp) or der(12) (937 bp) fusion junctions. ‘Chr7’ indicates an unedited region of chromosome 7 used as control to confirm the presence of sufficient DNA template (735 bp). The lowest cell number at which a band is visible marks the limit of detection.


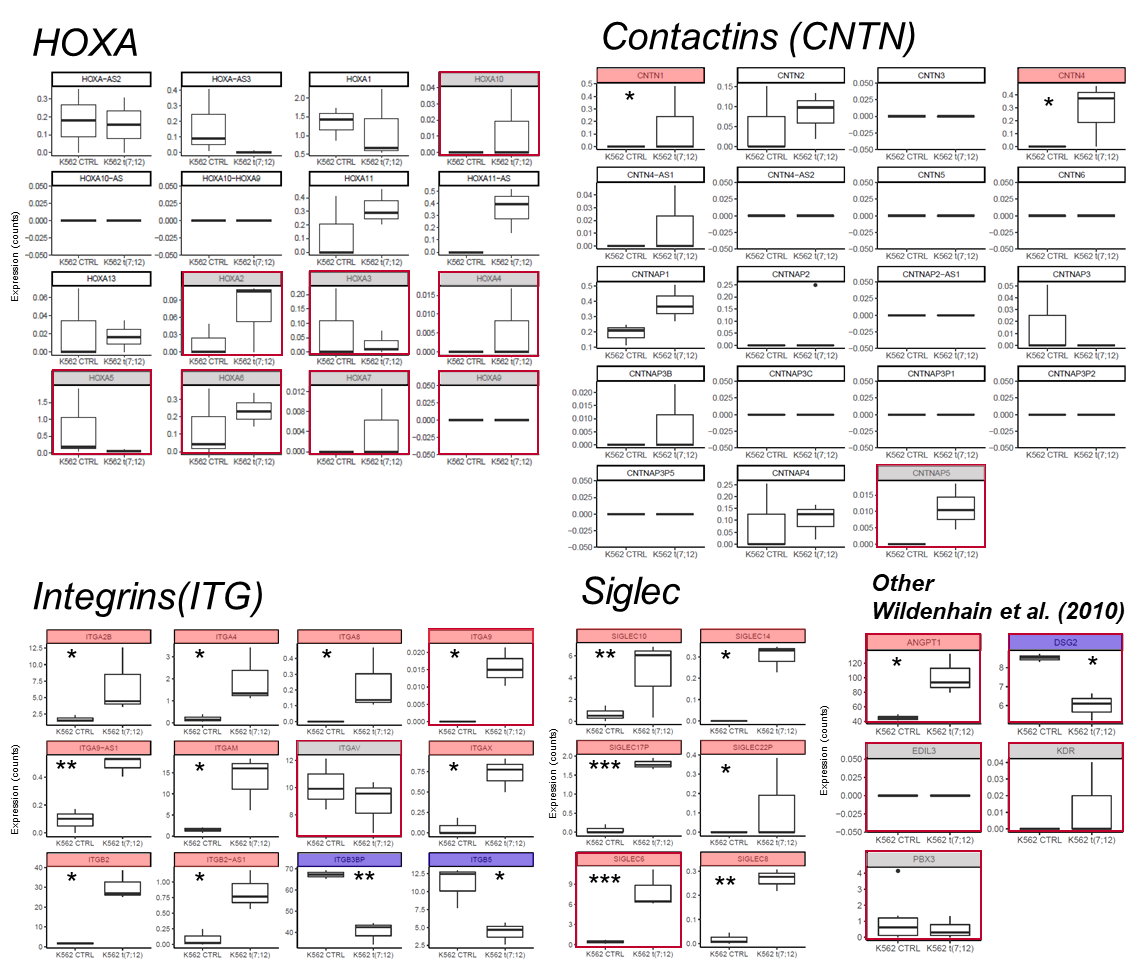


*
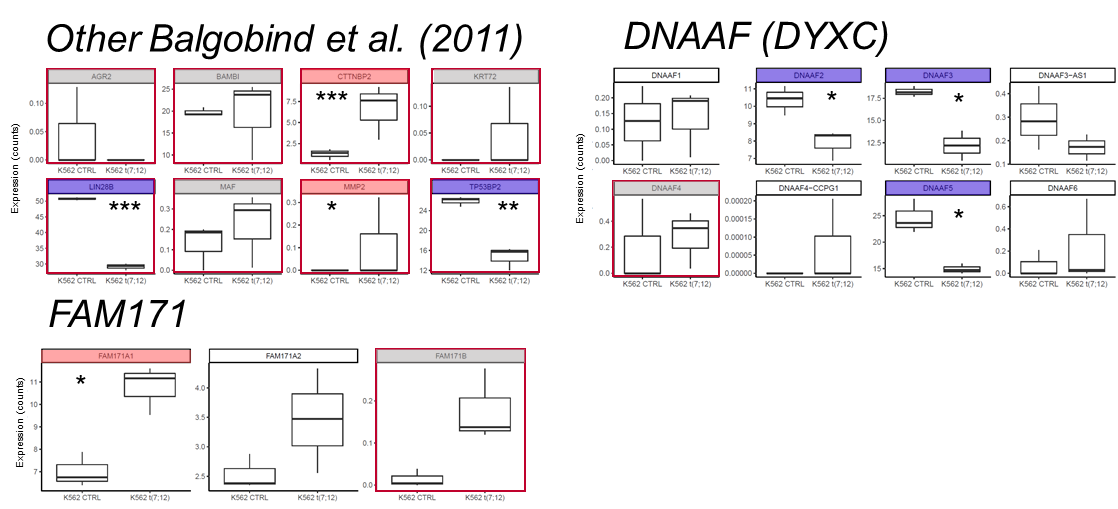
*

**Supplementary Figure 6 – Expression differences in K562-t(7;12) compared to K562 control of known t(7;12)-associated genes.** Gene expressions were extracted from RNA sequencing counts, and grouped by gene families. Red squares around the plot indicate a gene previously reported by Wildenhain et al. (2010) or Balgobind et al. (2011) as specific for the t(7;12) subtype. Grey shading indicates that the gene was not significantly dysregulated in K562-t(7;12) determined by T-test, while red indicates a significant upregulation, and blue a significant downregulation. Asterisks symbolise p value thresholds of 0.05 (*), 0.001 (**), 0.0001 (***), and 0.00001 (****).


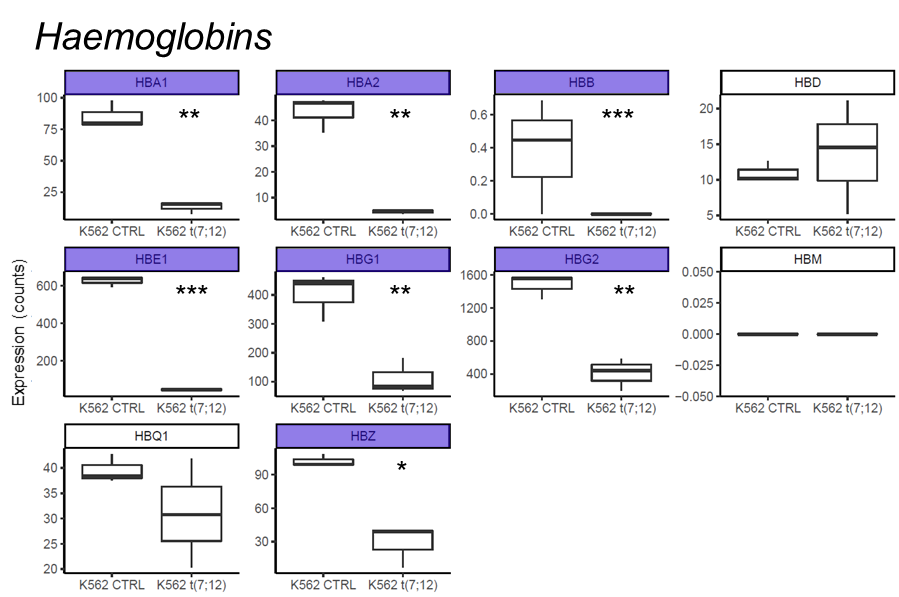


**Supplementary Figure 7 – Differentially expressed haemoglobin genes in K562-t(7;12).** Gene expressions were extracted from RNA sequencing counts of K562-t(7;12) and K562 control. Blue shading indicates a significant downregulation determined by T-test. Asterisks symbolise p value thresholds of 0.05 (*), 0.001 (**), 0.0001 (***), and 0.00001 (****).

**Supplementary Tables**

**Supplementary Table 1 – gRNA sequences**

| **NAME** | **SEQUENCE (5’-TO-3’)** | **PAM** |
| --- | --- | --- |
| 7q36 gRNA 4 | ATGGTCAGTCGTGCTCGGAA | AGG |
| ETV6 gRNA 4 | GAGGAAGCGTAACTCGGCAC | TGG |

**Supplementary Table 2 – PCR primer sequences**

| **PRIMER NAME** | **SEQUENCE 5’-TO-3’** |
| --- | --- |
| 7q36 4 F | GTTTGGGGAAACAATGGCCC |
| 7q36 4 R | GAGGCTACCAAGTGAAGCGT |
| Chr7 F | GCCACATCATCCAATGCTGG |
| Chr7 R | CTCCTGGGCCACAAGACAAT |
| Der7 F-3 | CTGCACTGATGAAGCCGATG |
| Der12 R-4 | GAAACAAGGAACAGGAACCTCAAA |
| Der7 R-4 | GGCTGTTTTCAACGATGGCA |

**Supplementary Table 3 – FISH probes ideograms**

| **NAME** | **IDEOGRAM** | **FLUOROPHORES** | **MANUFACTURER** |
| --- | --- | --- | --- |
| **XL t(7;12) MNX1/ETV6** | 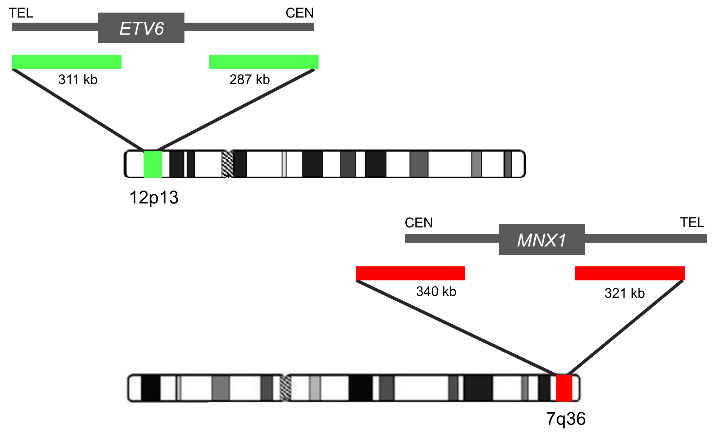 | FITC (ETV6)  Cy3 (MNX1) | Metasystems |
| **Dual-colour ETV6** | 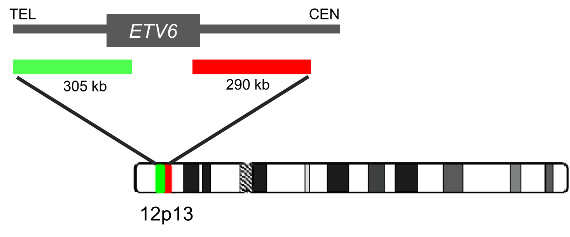 | FITC (ETV6 5’)  Cy3 (ETV6 3’) | Metasystems |
| **RP5-1121A15** | 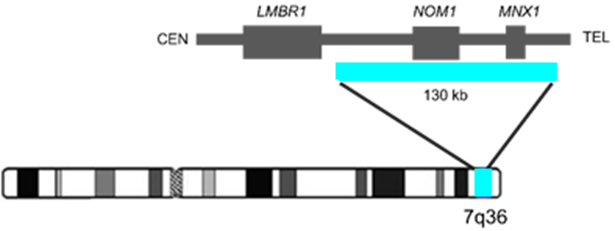 | Cy5  (cyan false colour) | PAC-clone |

**Supplementary Table 4 – qPCR primer sequences**

| **GENE** | **FORWARD PRIMER 5’-TO-3’** | **REVERSE PRIMER 5’-TO-3’** | **REFERENCE** |
| --- | --- | --- | --- |
| *HPRT1* | CCTGGCGTCGTGATTAGTGAT | TCGAGCAAGACGTTCAGTCC | Arede *et al.*, 2022 (2) |
| *HBB* | AGGAGAAGTCTGCCGTTACTG | CCGAGCACTTTCTTGCCATGA | Arede *et al.*, 2022 (2) |
| *EPOR* | TGGCACATAGCGAACATTCCA | GGCTGGGAAGAGAATGCTGATT | This study |
| *TAL1* | TGATGGTCCCCACACCAAAG | AAAGGCCCCGTTCACATTCT | This study |
| *MNX1* | GTTCAAGCTCAACAAGTACC | GGTTCTGGAACCAAATCTTC | Gulino *et al.*, 2021 (3) |

**Supplementary Table 5 – 122-signature gene list**

| **122 - signature** |  |  |  |  |
| --- | --- | --- | --- | --- |
| *MNX1*  *TTC39C*  *CNTNAP5*  *ACOT9*  *TLR2*  *WIPF1*  *MNX1-AS1*  *CTTNBP2*  *OGFRL1*  *GALNT18*  *PRB2*  *MEIS2*  *MYD88*  *LBR*  *IRAK3*  *CYP2C18*  *POU3F3*  *SLITRK5*  *UNC13C*  *SGMS2*  *SIGLEC8*  *RGS18*  *TFEC*  *DMRTA1*  *PRB1* | *SH3KBP1*  *CSN1S1*  *RORB*  *PITPNM3*  *PHACTR2*  *TRPC4*  *KRT2*  *MOCS2*  *FOXB1*  *TMEM217*  *PTGER2*  *FGF7*  *RAPGEF4*  *CXCR4*  *ISOC1*  *COBL*  *KIF13B*  *MYOF*  *TMX4*  *FAM83B*  *SLC4A7*  *BOC*  *TFAP2A*  *LIN28B*  *ZNF474* | *NUDT4*  *TES*  *ASAP1*  *DISP3*  *SPSB4*  *KIAA1217*  *CREB5*  *BAMBI*  *MBD2*  *MCTP1*  *NLRP11*  *OR7E38P*  *TARS3*  *NECTIN2*  *CXCL17*  *TENT4B*  *DHRS7*  *TEC*  *PIK3CG*  *TSPO*  *EVI2B*  *ABCA8*  *TM6SF1*  *RAMP2-AS1*  *INPPL1* | *RCBTB2*  *WSB1 ACP3*  *PON2*  *ENOSF1*  *TP53BP2*  *ENOX1*  *METTL9*  *CDX2*  *FER*  *ST3GAL6*  *FOXA2*  *PTPN7*  *FSIP2*  *DENND10P1*  *PHF12*  *ZNF114*  *CCPG1*  *GRIK1-AS1*  *ZNRF1*  *OR7E11P*  *ADARB2*  *FAM126A*  *ROBO2*  *PELI2*  *ADA2* | *STK39*  *NUDT4B*  *ABCA6*  *B3GALNT1*  *KITLG*  *CAV3*  *CSGALNACT2*  *KCNIP1*  *PPM1L*  *PPIL6*  *KDR*  *CPQ*  *GNB3*  *COL6A3*  *GPR137B*  *NMNAT3*  *CYP2C9*  *PRSS1*  *PPFIA3*  *ATF7IP2* |

**Supplementary Table 6 – 177-signature gene list**

| **177 - signature** |  |  |  |  |
| --- | --- | --- | --- | --- |
| *COL6A3*  *COL6A2*  *COL6A1*  *DNMT3B*  *CTBS*  *NLRP11*  *TLR2*  *HOXC-AS3*  *RARA-AS1*  *MAF*  *DHRS7*  *CCDC71L*  *WNT5B*  *PAPSS1*  *NCOA7*  *NETO1*  *NRN1*  *SH3BGRL2*  *SPRR2G*  *HERPUD2*  *FKBP9*  *ERMP1*  *FER*  *STAR*  *SGMS2*  *TARS3*  *LONRF3*  *PIK3C2B*  *PKM*  *MEGF9*  *SH2D1A*  *ECHDC3*  *FAM83B*  *LRMDA*  *CNTNAP5*  *GALNT18*  *SLITRK5* | *CYP2C18*  *SPSB4*  *RAPGEF4*  *LINC00887*  *ZNF474*  *TFAP2A*  *EVI2B*  *BTG3*  *TLE4*  *RASAL3*  *HEXA*  *CSRNP3*  *ASL*  *SFTPA2*  *SFTPA1*  *MYD88*  *TES*  *YPEL2*  *SIGLEC17P*  *MEIS2*  *LBR*  *RAB13*  *NDRG1*  *ST3GAL6*  *PDZD2*  *SIGLEC8*  *SIGLEC6*  *RGS18*  *PTGER2*  *HOXC9*  *LINC02573*  *PGLYRP4*  *PGLYRP3*  *CDIP1*  *PRB2*  *PRB1*  *ZNF366* | *TSPO*  *P3H3*  *NLRC3*  *FTCDNL1*  *AGR2*  *PI16*  *POU3F3*  *TMX4*  *FOXB1*  *EFCC1*  *LINC00299*  *LINC00298*  *ACOT9*  *DAP*  *INPPL1*  *AGA*  *TMEM92*  *ADGRL1*  *TFEC*  *LINC01694*  *IRAK3*  *SH3KBP1*  *LINC00240*  *PPP1R13L*  *TAS2R10*  *WDFY1*  *ACVR1B*  *ADGRG5*  *BTBD9*  *NUDT4*  *KRT74*  *KRT73*  *KRT72*  *ABCD3*  *WASIR2*  *WASIR1*  *MNX1* | *EVL*  *STARD9*  *C9orf72*  *LRRK1*  *OGFRL1*  *HLX*  *FAM30A*  *KDR*  *ANO2*  *ADGRA3*  *ZBTB20*  *DSG2*  *NUDT4B*  *PDE6G*  *FAM171B*  *UNC13C*  *ADA2*  *LIN28B*  *ATF7IP2*  *LRRC28*  *QSOX1*  *BOC*  *ISOC1*  *BAMBI*  *OAZ2*  *GSTA4*  *TWSG1*  *ABCA9*  *ABCA6*  *CDCP1*  *LPIN1*  *RASGEF1A*  *CRYZL2P*  *H2BC4*  *MAPK10*  *KIF13B*  *HPGD* | *SLC4A7*  *LINC01016*  *RAMP2-AS1*  *LTK*  *LTB*  *SPRY2*  *PIK3CG*  *DMRTA1*  *MNX1-AS1*  *VWF*  *ARSD*  *TTC39C*  *CRTAP*  *KRT2*  *WIPF1*  *C2CD2*  *JAM3*  *NETO1-DT*  *SIGLECL1*  *GIMAP6*  *CSGALNACT2*  *TFAP2A-AS1*  *CTTNBP2*  *PRNP*  *MFGE8*  *TP53BP2*  *CYP2C9*  *PRSS2*  *MMP9* |

**Methods**

1. **Cell cultures**

The K562 leukaemia cell line was grown in RPMI1640 (Gibco, Paisley, UK), 10% foetal bovine serum (FBS) (Gibco) and 1% penicillin/streptomycin antibiotics (100 U/mL/100 μg/mL, Gibco). Peripheral blood mobilised CD34+ haematopoietic stem and progenitor cells (HSPCs) were obtained from UCL, Institute of Child Health, Infection, Immunity and Inflammation Programme (London, UK), purchased from commercially available apheresis products. CD34+ HSPCs were cultured in StemSpan medium (StemCell Technologies, Cambridge, UK) supplemented with 100 ng/ml SCF, 100 ng/ml FLT3-L, 20 ng/ml TPO, and 20 ng/ml IL-3 (Peprotech, London, UK). Cell lines were tested for Mycoplasma contamination using MycoSensor PCR Assay Kit (Agilent, Didcot, UK).

1. **CRISPR/Cas9 editing and clone selection**

Sequences for human *MNX1* (accession number NC_000007.14) and *ETV6* (accession numbers NC_000012.12) were retrieved for gRNA design by the Invitrogen TrueDesign Genome Editor tool (threshold at 1 nucleotides of mismatch tolerance). gRNA sequences and the adjacent PAM sequence used in this study are reported in **Supplementary Table 1**. GeneArt Precision gRNA Synthesis Kit (Invitrogen, Inchinnan, UK) was used to synthesise customised gRNAs via in vitro transcription (IVT). The transfection of the gRNA and Cas9 endonuclease was achieved by assembly and delivery of an RNP complex via electroporation using the Neon Transfection System Kit (Invitrogen). The RNP complex was assembled at 1:2 molar ratio of 30.6 pmol of TrueCut™ Cas9 Protein v2 (Invitrogen) to 61.2 pmol synthesised gRNA, and incubated at 37°C for 15 minutes prior to electroporation. Electroporation was carried out using the following settings: 1450 V, 10 ms, 3 pulses. K562-Ctrl was obtained by electroporation of Cas9 protein only.

K562 clones harbouring the translocation were isolated by single cell cloning by limiting dilution and PCR screening. An initial t(7;12)-harbouring clone (named A10 in **Supplementary** **Figure 3A**), detected by the presence of fusion junction by PCR, was isolated and further subcloned. Three subclones were used for all experiments and RNA sequencing, in which the t(7;12) was further confirmed by PCR (**Supplementary Figure 3B**). The K562-Ctrl was also subjected to single cell cloning, from which 3 clones were selected.

1. **Amplification of t(7;12) fusion junctions by polymerase chain reaction (PCR)**

PCR on genomic DNA was performed using High-Fidelity Phusion polymerase (Invitrogen) using manufacturer’s instructions. PCR primers (**Supplementary Table 2**) were designed using Primer-BLAST (NCBI). The complete genomic sequences of human MNX1 and ETV6 were obtained from the NCBI Gene and Nucleotide Database with accession numbers NC_000007.14 for *MNX1* and NC_000012.12 for *ETV6*. The amplified fragments of the correct estimated size for K562 Mut3 were excised, purified from the gels, and sequenced (**Error! Reference source not found.**).

1. **Fluorescence in situ hybridisation (FISH)**

Preparation of metaphase chromosomes was performed by colcemid treatment (0.05 μg/mL) as described (Federico *et al.*, 2019) and fixed in methanol:acetic acid. FISH was performed using commercially available (MetaSystems, Altlussheim, Germany) and PAC-derived probes (**Supplementary Table 3**). PAC-derived probes were extracted from bacterial cultures and fluorescently labelled by nick translation (Roche, Mannheim, Germany). Following hybridisation and washes according to published protocols (Federico *et al.*, 2019), images were captured using a Leica DM4000 fluorescence microscope (Leica Microsystems, Wetzlar, Germany). A minimum of 200 nuclei were captured for analysis. Representative images of K562 control and K562-t(7;12) nuclei hybridised with the probe combinations and the interpretation of signal patterns are shown in Error! Reference source not found..

1. **RNL analysis**

Radial nuclear location (RNL) was calculated using 2D analysis of nuclei hybridised by FISH as previously described (4). The RNL is defined as a numerical value corresponding to the positioning of a FISH signal as the ratio of the nuclear radius. RNL ranges from 0 to 1, with 0 marking the interior and 1 marking the outer extreme. A minimum of 200 nuclei per condition are analysed; RNL is expressed taking into consideration the median value of all signals ± confidence interval, with a 0.650 and lower defining the nuclear interior (4).

1. **Real-time quantitative PCR (qPCR)**

Total RNA was extracted using the Monarch Total RNA Miniprep Kit (New England Biolabs, Hitchin, UK), from which complementary DNA (cDNA) was synthesised by reverse transcription using High-Capacity RNA-to-cDNA Kit (Applied Biosystems, Waltham, US). qPCR was performed using FastGene 2x IC Green Universal qPCR Mix (fluorescein) (Nippon Genetics, Düren, Germany) for each gene with primers listed in **Supplementary Table 4**. Differential gene expression was calculated using the ΔΔCt method. *HPRT-1* was used as endogenous reference gene.

1. **Colony forming assay**

Cells were plated onto semi-solid methylcellulose for colony-forming assays (CFC), in Methocult H4434 Classic (StemCell Technologies, Cambridge, UK). Cells were first suspended in IMDM (Gibco) + 20% FBS (Gibco) at the desired concentration (1000 – 10000 cells / plate). Plates were incubated at 37°C and 5% CO2 for 10-12 days, when colonies were scored.

1. **Long-Term Culture Initiating Cell Assay (LTC-IC)**

The MS5 stroma cells were plated in a 96-well plate at a density of 30 000 cells per plate. 45 000 CD34+ HSCPs were added to each well in 100 μl of H5100 Myelocult (StemCell Technologies), 1 μM hydrocortisone (StemCell Technologies), 20 ng/ml of IL-3, TPO, G-CSF, and SCF (Peprotech). The medium was replaced weekly and the assay was conducted for 4 weeks. At the end of the 4th week, 100 μl of Methocult H4434 (StemCell Technologies) was added to each well, and colonies were scored after 10 days. If colonies were present, the methylcellulose-based culture was dissociated by extensive resuspension and washes in PBS and cells were replated onto MS5 stroma for a further 5 week period, followed by methylcellulose addition.

1. **Erythroid differentiation assay**

K562 cells were subjected to erythroid differentiation by induction by DMSO (Fisher Scientific, Paisley, UK) over a period of 6 days, as described (2). 100 000 cells were seeded. At E0, 1.5% DMSO was added onto the culture medium (RPMI + 10% FBS + 1% P/S). RNA was collected at E0, E2, and E6 for subsequent qPCR analysis of erythroid gene markers *HBB, EPOR,* and *TAL1*.

1. **RNA sequencing analysis**

RNA sequencing was performed by Macrogen Europe BV (Amsterdam, Netherlands). A minimum of 1 μg (20 ng/μl) of high-quality total RNA (extracted using Monarch Total RNA Miniprep Kit, NEB) was supplied for sequencing. Macrogen Europe BV constructed libraries using Illumina Truseq Stranded Total RNA library preparation with Ribozero rRNA depletion, and performed sequencing was performedon a Novaseq 6000 platform, at 50M paired-end reads per sample. RNA-seq results in the form of fastq raw reads were analysed with the open source software package of the Tuxedo Suite. Tophat2 with Bowtie2 were used to map paired-end reads to the reference Homo sapiens genome build GRCh38 (5,6). GENCODE38 (7) was used as the reference human genome annotation. Aligned reads were filtered by quality using samtools (8) with a minimum selection threshold set at 30. Transcript assembly and quantification was achieved using Cufflinks (9). Differential expression between sample and control was performed by collapsing technical replicates for each condition and the use of the Cuffdiff tool (9). The differential expression was expressed in the form of log2 fold change between sample and control, and deemed statistically significant by a lower p value of 0.05 and false discovery rate (FDR) of 0.1.

1. **Gene Ontology analysis**

Gene ontology (GO) analysis was performed in ExpressAnalyst (available at [www.expressanalyst.ca](https://www.expressanalyst.ca)) using PANTHER Biological Process (BP) and Molecular Function (MF) repositories. GO terms and pathways were filtered by p value and false discovery rate (FDR) with a cut-off of ≤ 0.05 for meaningful association.

1. **Flow cytometry staining**

Expression of cell surface markers was determined by staining and analysed by flow cytometry. Cells were pelleted by centrifugation and resuspended in basic flow cytometry buffer PFE (PBS, 2% FBS and 0.5 mM EDTA). Human c-KIT (CD117) antibody (clone 2B8) labelled in APC was purchased from Biolegend (Uithoorn, Netherlands) and used at a dilution of 1:30.

1. **Statistical analysis and visualisation**

Statistical significance was calculated by two-tailed Student's t-test on available replicates (minimum n=3). Variance is represented in barcharts by error bars of ± standard deviation (SD). A p value ≤ 0.05 was considered statistically significant, unless otherwise stated. Statistical significance is symbolised as: p value ≤ 0.05 (*), 0.001 (**), 0.0001 (***), and 0.00001 (****). Calculations were performed in R studio version 4.1.0. Graphs were generated in Microsoft Excel 2013 and in R studio 4.1.0 using the libraries ggplot2 (v 3.3.5) and ggrepel (v 0.9.1). Multiplex Venn diagrams were generated on VennPainter (10).

1. **Patient signature methods and data availability**

Clinical phenotype and expression data (in counts units) were extracted from the Therapeutically Applicable Research to Generate Effective Treatments (TARGET, <https://ocg.cancer.gov/programs/target>) available in the GDC TARGET-AML cohort last accessed on 3^rd^ June 2022 from the University of California Santa Cruz (UCSC) Xena public repository (11). Microarray data in RMA units of expression for the TARGET AML cohort, including normal bone marrow data, was downloaded from the NCI TARGET Data portal. Microarray data in RMA units of expression for paediatric AML from Balgobind *et al.* (12) (under accession number GSE17855) was downloaded from GEO Accession Viewer. Data normalisation across arrays was achieved by Training Distribution Matching (TDM) (13) and any remaining batch effects were removed by Limma removeBatchEffect. Differential expression analysis was performed using Limma. To construct the gene signatures, all differentially expressed genes for each comparison below a p value of 0.01 were included and intersected accordingly using Venn diagrams on VennPainter (10).

1. **Gene Set Enrichment Analysis (GSEA)**

Custom gene signatures were used as gene sets for GSEA analysis on the GSEA software v4.2.3 on the RNA sequencing expression values in counts units of K562-t(7;12) against K562 control. GSEA was ran in 10 000 permutations on gene set using the weighted Signal2Noise metric. Enrichment plots and leading edge heatmaps were included as generated from the GSEA software.

**Supplementary References**

(1) Kamel YM, Naiel A, Alshehri A, Vetter M, Saccone S, Anderson R, et al. Fluorescence in situ hybridisation assays designed for del (7q) detection uncover more complex rearrangements in myeloid leukaemia cell lines. 2014.

(2) Arede L, Foerner E, Wind S, Kulkarni R, Domingues AF, Giotopoulos G, et al. KAT2A complexes ATAC and SAGA play unique roles in cell maintenance and identity in hematopoiesis and leukemia. Blood advances 2022;6(1):165-180.

(3) Gulino GM, Bruno F, Sturiale V, Brancato D, Ragusa D, Tosi S, et al. From FISH to Hi-C: The Chromatin Architecture of the Chromosomal Region 7q36. 3, Frequently Rearranged in Leukemic Cells, Is Evolutionary Conserved. International Journal of Molecular Sciences 2021;22(5):2338.

(4) Federico C, Cantarella CD, Di Mare P, Tosi S, Saccone S. The radial arrangement of the human chromosome 7 in the lymphocyte cell nucleus is associated with chromosomal band gene density. Chromosoma 2008;117(4):399-410.

(5) Kim PG, Albacker CE, Lu YF, Jang IH, Lim Y, Heffner GC, et al. Signaling axis involving Hedgehog, Notch, and Scl promotes the embryonic endothelial-to-hematopoietic transition. Proc Natl Acad Sci U S A 2013 Jan 8;110(2):E141-50.

(6) Langmead B, Salzberg SL. Fast gapped-read alignment with Bowtie 2. Nature methods 2012;9(4):357-359.

(7) Frankish A, Diekhans M, Ferreira A, Johnson R, Jungreis I, Loveland J, et al. GENCODE reference annotation for the human and mouse genomes. Nucleic Acids Res 2019;47(D1):D766-D773.

(8) Li H, Handsaker B, Wysoker A, Fennell T, Ruan J, Homer N, et al. The sequence alignment/map format and SAMtools. Bioinformatics 2009;25(16):2078-2079.

(9) Trapnell C, Roberts A, Goff L, Pertea G, Kim D, Kelley DR, et al. Differential gene and transcript expression analysis of RNA-seq experiments with TopHat and Cufflinks. Nature protocols 2012;7(3):562-578.

(10) Lin G, Chai J, Yuan S, Mai C, Cai L, Murphy RW, et al. VennPainter: a tool for the comparison and identification of candidate genes based on Venn diagrams. PloS one 2016;11(4):e0154315.

(11) Goldman MJ, Craft B, Hastie M, Repečka K, McDade F, Kamath A, et al. Visualizing and interpreting cancer genomics data via the Xena platform. Nat Biotechnol 2020;38(6):675-678.

(12) Balgobind BV, Hollink IH, Arentsen-Peters ST, Zimmermann M, Harbott J, Beverloo HB, et al. Integrative analysis of type-I and type-II aberrations underscores the genetic heterogeneity of pediatric acute myeloid leukemia. Haematologica 2011 Oct;96(10):1478-1487.

(13) Thompson JA, Tan J, Greene CS. Cross-platform normalization of microarray and RNA-seq data for machine learning applications. PeerJ 2016;4:e1621.
